# Auracast and hearing devices: a scoping review on connectivity and accessibility for individuals with hearing loss in public environments

**DOI:** 10.1590/2317-1782/e20250241en

**Published:** 2026-07-06

**Authors:** Lucas Marini Gonçalves, Anderson Vinícius de Moraes Ortega, Thais Corina Said de Angelo, Adriane Lima Mortari Moret, Natália Barreto Frederigue Lopes, Regina Tangerino de Souza Jacob

**Affiliations:** 1 Department of Speech-Language Pathology and Audiology, Bauru School of Dentistry – FOB, Universidade de São Paulo – USP, Bauru (SP), Brazil.

**Keywords:** Assistive Technology, Hearing Aids, Accessibility, Inclusive Education, Bluetooth Communication

## Abstract

**Purpose:**

To map and synthesize the available evidence on the use of Auracast technology in hearing aids (HA), in the context of auditory accessibility in public environments.

**Research strategies:**

This scoping review followed the Joanna Briggs Institute (JBI) and PRISMA-ScR guidelines. The PCC framework was adopted (Population: individuals with hearing loss using HA; Concept: Auracast technology; Context: public environments). The search strategy was based solely on the keyword “Auracast” and applied to a defined time window from June 2022 (the official launch of Auracast) to April 2025. Information sources included scientific databases (PubMed, Scopus, IEEE Xplore, Dimensions Analytics) and gray literature sources (Google Scholar, websites of HA manufacturers, professional associations, and institutional repositories such as OSF and OpenGrey).

**Selection criteria:**

Study selection and data extraction were conducted independently and blindly by two reviewers, with disagreements resolved by consensus.

**Data analysis:**

A total of 348 records were identified. After removing duplicates and applying eligibility criteria, 17 studies were included in the final synthesis.

**Results:**

The available evidence suggests that Auracast could improve accessibility and autonomy in public settings, particularly when integrated with HA. However, most studies were technical or descriptive, lacking robust clinical evaluations.

**Conclusion:**

Auracast is an emerging innovation in auditory accessibility. Nevertheless, further empirical studies are needed to assess its effectiveness in educational, healthcare, and transportation settings. Large-scale adoption will depend on technical, financial, and social factors, especially in low-infrastructure contexts.

## INTRODUCTION

Since its introduction in electronic hearing aids (EHAs), hereinafter referred to as hearing aids, in 2005, Bluetooth technology has played a fundamental role in the connectivity of individuals with hearing loss, enabling the direct transmission of audio to devices such as cell phones, tablets, and computers^(1)^. Recently, an innovation has emerged in this field: Bluetooth Low Energy (LE) Audio with Auracast, an advancement designed to integrate wireless audio technologies and enhance hearing accessibility. Similar to how Wi-Fi networks work, this technology allows users with compatible devices to connect to audio streaming systems in Auracast-equipped environments^(2)^.

Auracast technology represents a significant advancement in audio connectivity in public spaces. Compatible devices, such as TVs, mobile phones, laptops, and microphones, can transmit audio directly to receiving devices, including hearing aids, headphones, and smartwatches^(3)^. This feature is particularly relevant for individuals with hearing loss, as it enables a clearer and more intelligible sound experience, reducing auditory effort and fatigue associated with communication in noisy or reverberant environments. Furthermore, the technology enables large-scale audio transmission, allowing its application in classrooms, conference centers, stadiums, churches, and other locations that require enhanced hearing accessibility^(4)^.

The development of assistive technologies has been essential to promoting inclusion and improving communication for people with hearing impairments. The Auracast network, by offering wireless audio transmission without restrictions on the number of connected devices, presents a relevant technological alternative for democratizing access to sound information. Its implementation can transform auditory accessibility in public and private spaces, representing a substantial advance in wireless connectivity for this population^(5)^.

Given this scenario, this study aims to map and synthesize the available scientific evidence on Auracast technology, based on Bluetooth Low Energy, applied to hearing aids, focusing on its contribution to hearing accessibility in public environments. Considering the recent launch and rapid dissemination of Auracast, the hypothesis is that there are already technical and scientific records demonstrating its potential application in hearing aids to promote hearing accessibility in public environments, even though clinical studies are scarce.

With this approach, it is expected to contribute significantly to the evolution of wireless connectivity in hearing devices, providing theoretical and practical support to academia, hearing healthcare professionals, and public policy makers^(6)^.

## METHOD

This Scoping Review was developed based on the methodology of the Joanna Briggs Institute (JBI) Reviewers’ Manual and follows the PRISMA-ScR (Preferred Reporting Items for Systematic Reviews and Meta-Analyses Extension for Scoping Reviews) guidelines, ensuring transparency and reproducibility^(7,8)^. The study protocol was submitted for evaluation and registration on the Open Science Framework (OSF) platform^(9)^.

### Eligibility criteria

The eligibility criteria were defined based on the PCC (Population, Concept, and Context) model, being Population - individuals with hearing loss who use hearing aids; Concept - Auracast technology applied to hearing aids; Context - hearing accessibility in public environments.

### Inclusion and exclusion criteria

The inclusion criteria were defined based on the PCC model, considering publications that addressed individuals with hearing loss who use hearing aids, and that explicitly discussed Auracast technology in the context of hearing accessibility in public environments, such as schools, cultural centers, churches, transportation stations, and other collective spaces. Documents published between June 2022 (the official launch date of Auracast) and April 2025, available in full text, freely accessible, and written in Portuguese, English, or Spanish were included. The review encompassed different types of publications, such as scientific articles, dissertations, theses, technical reports, institutional publications, and proceedings of academic events.

Publications that did not mention the term “Auracast” or addressed it superficially, without a direct relationship to the application of the technology in hearing aids, were excluded. Documents that focused exclusively on other Bluetooth-based assistive technologies without reference to Auracast, duplicate publications, documents unavailable in full, or written in languages ​​not covered were also excluded.

### Research sources and search strategy

To identify relevant sources, the term “Auracast” was used exclusively as a keyword in all information sources. Although controlled descriptors (such as MeSH or DeCS) and free terms associated with the broader concept of assistive technologies were not used, this choice is justified by the specific and delimited focus of the review on Auracast technology, a distinctive and recent name with a well-defined occurrence in publications.

The searches were conducted in the following databases: PubMed, BVS, SciELO, IEEE Xplore, ACM Digital Library, Cochrane Library, and Scopus. Since the number of results in these databases was limited, the search was expanded to the Dimensions Analytics platform, which aggregates various sources of scientific, technical, and institutional output, and to Google Scholar, considered a broad source of information, although not formally classified as a database.

Additionally, a manual search was conducted to locate both white and grey literature. Institutional repositories (such as OSF and OpenGrey), websites of hearing aid manufacturers (such as GN Hearing and Sonova), and relevant industry associations (such as the Bluetooth Special Interest Group – SIG and the Hearing Loss Association of America) were consulted. This search was performed using structured navigation and specific terms in internal search engines or general search engines.

The Web of Science database was not included in the search strategy, as the methodological design prioritized databases with greater simultaneous relevance to the areas of health, engineering, and applied technology, such as Scopus, IEEE Xplore, ACM Digital Library, and Dimensions Analytics. These platforms offer broad multidisciplinary coverage and strong indexing of technical and technological innovation publications, considered particularly relevant to the Auracast theme. Therefore, the strategy adopted sought to maximize sensitivity in identifying technical, scientific, and institutional records related to the technology, while maintaining consistency with the emerging and interdisciplinary nature of the investigated object.

Technical documents, guides, reports, institutional communications, scientific articles, dissertations, theses, and events proceedings were considered eligible. Two independent reviewers screened the materials based on the established criteria, prioritizing publications that addressed the application of Auracast in hearing aids, with a focus on hearing accessibility.

This approach sought to circumvent the scarcity of peer-reviewed evidence expected given the recent official introduction of Auracast by the Bluetooth SIG in June 2022. Thus, the incorporation of technical and institutional literature was essential to ensure the comprehensiveness and timeliness of the review.

### Data extraction and storage

An automated bibliographic reference manager was not used in this study. The organization and control of sources were carried out manually using electronic spreadsheets, which allowed systematic screening, categorization and exclusion of duplicates. The elimination of duplicate references was carried out through direct comparison of titles, authors, years and access links, with cross-review between reviewers. Although tools such as Zotero or Mendeley can facilitate this process, direct and personalized control was used due to the manageable volume of records and the specificity of the term “Auracast”, which allowed precise management of publications, without compromising the transparency and reproducibility of the review.

The screening process was performed in three phases: reading titles, reading abstracts, and complete reading of the selected texts.

All phases of the selection and extraction process were carried out independently by two reviewers. To ensure blinding, the records were organized in spreadsheets without identifying authors, institutions or journals. Access to full texts was structured by code, and screening sheets were shared separately with each reviewer. Blinding was also maintained in the data extraction step, which followed a pre-defined protocol with standardized fields.

In case of disagreement between the reviewers - both in the screening phase (step 1: reading titles and abstracts; step 2: complete reading) and in the extraction phase - the conflicts were discussed in a joint meeting. When there was no consensus, the decision was submitted to a third reviewer with experience in the topic, who evaluated the inclusion criteria in light of the previously defined protocol.

The extracted data were analyzed by two authors, being categorized into: Authors (year), Country, Classification of Publication, and Observations Related to Auracast.

### Analysis and presentation of results

Data were analyzed descriptively and presented through figures and tables, as recommended by PRISMA-ScR^(8)^.

## RESULTS

### Review and selection of results

The initial search identified a total of 348 records, distributed in the following sources: Google Scholar (296), Dimensions Analytics (44), IEEE Xplore (2), PubMed (2), Scopus (4), SciELO (0), VHL (0), ACM Digital Library (0), Cochrane Library (0). The Dimensions database was used as an alternative to expand the coverage of relevant technical and scientific literature on Auracast, given the reduced number of results in conventional databases. Google Scholar was included as a source of information to cover materials not indexed in traditional databases, as recommended for reviews involving gray literature.

After manually removing seven duplicate records, 341 publications were submitted to the screening phase. At this phase, titles and abstracts were evaluated based on the PCC criteria: 322 records were excluded because they did not address Auracast technology in the context of hearing accessibility, because they were different technologies or because they referred to technical areas unrelated to the target population (individuals with hearing loss using hearing aids) or to the context of public environments.

**Figure 1:**
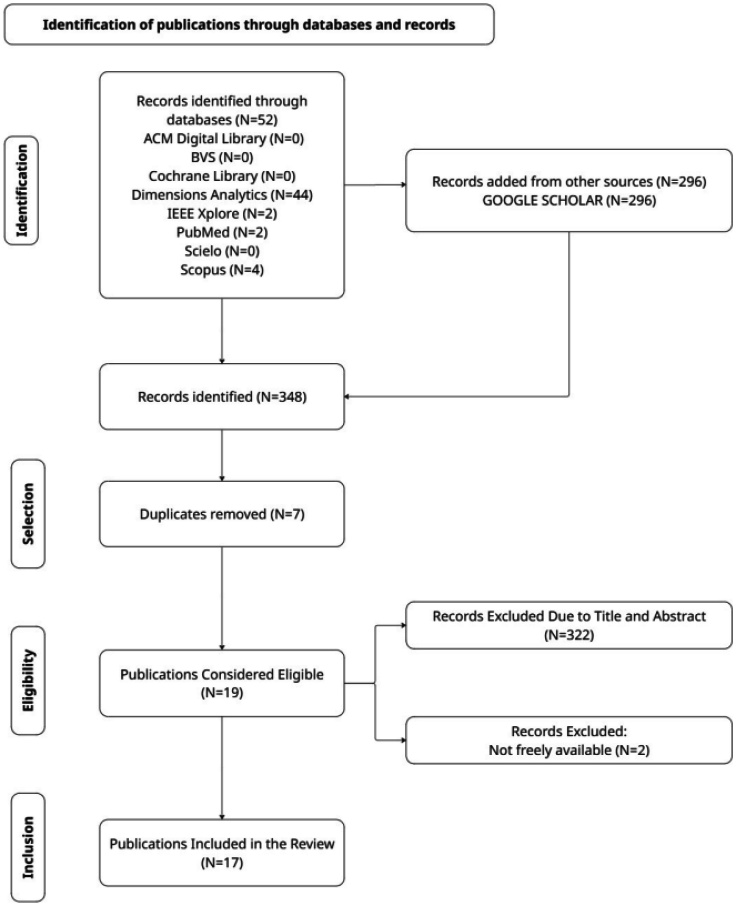
Flowchart of the selection process for publications included in the review, according to the identification, screening, eligibility, and inclusion phases, based on the PRISMA-ScR model

Two of the 19 publications selected for complete reading were excluded due to the unavailability of free access to the full text. Although an attempt was made to contact the authors, there was no response within the deadline set for the extraction. Therefore, 17 documents met all eligibility criteria and were included in the final synthesis. The complete flowchart of the selection process of the studies is shown in Figure 1, according to the PRISMA-ScR model guidelines.

### Study characteristics

The 17 studies selected for this review were published between 2023 and 2025, in different formats - such as scientific articles, theses, technical documents, manufacturer announcements, and online dissemination content - and mostly in English, with one exception in French (also translated into Spanish) (Table 1). The diversity of sources highlights the growing interest in emerging technologies applied to hearing accessibility.

**Table 1:** Summary of results

**Authors**	**Country**	**Classification of publication**	**Objective of the study**	**Notes related to Auracast**
Al-Heeti^(10)^	USA	Technology article (online dissemination)	Announce the integration of Auracast support into Android, allowing hearing aids to connect directly to audio transmissions	Auracast is added to Android, allowing hearing aids to connect directly to audio transmissions such as public announcements, significantly improving hearing accessibility for users with hearing loss
Bailey^(11)^	Australia	Technology article (online dissemination)	Report the implementation of Auracast at the Sydney Opera House and discuss its advantages over traditional assistive listening systems	Auracast is pioneered in the Sydney Opera House, demonstrating its quick and affordable installation, providing high-quality audio without interference, overcoming limitations of assistive listening systems based on magnetic induction
Bruce et al.^(12)^	Canada/ India	Conference paper (ICASSP)	Analyze the development of the Bluetooth LE Audio standard with Auracast and discuss technical opportunities and challenges for its implementation as an assistive listening system	Auracast is presented as a technological evolution with the potential to replace traditional assistive listening systems by allowing stereo audio transmission with low latency and universal connection to hearing aids and headphones, in addition to supporting multiple simultaneous streams - such as different languages, sound mixes or specific sources in public environments. However, its adoption still requires solutions for volume calibration and acoustic delay compensation
Ceccato et al.^(13)^	France	Review article (scientific journal)	Describe the technical and clinical foundations of fitting conventional hearing devices, including auditory rehabilitation strategies	Auracast is presented as a promising technological resource, allowing simultaneous transmission of audio to multiple compatible hearing devices, with the potential to increase accessibility in public environments
Edwards et al.^(14)^	USA	Conference paper (EPIC Proceedings)	Investigate the experience of hearing aid users when connecting to PCs and the impact on accessible design decisions	Auracast is mentioned as a Bluetooth LE Audio feature with the potential to overcome technical connectivity barriers, allowing simultaneous and efficient pairing between hearing aids and PCs
Fabry^(15)^	USA	Professional disclosure article (ASHA Leader)	Explore technological innovations in hearing aids, with a focus on connectivity and audio transmission	Auracast is highlighted as an emerging technology that, by allowing audio transmissions to multiple devices simultaneously, promises to revolutionize the listening experience in public and private environments, although its implementation still depends on advances in infrastructure and device compatibility
Gasteiger et al.^(16)^	Austria / Italy	Conference paper (MILCOM)	Propose and validate an authentication mechanism (BACON) for Auracast transmissions in public environments vulnerable to attacks	Auracast is described as having potential for large-scale public applications, but its secure adoption still requires authentication solutions like BACON to mitigate vulnerabilities and ensure transmission integrity
GN Hearing^(17)^	Denmark / USA	Editorial note in a professional journal (Manufacturers News – The Hearing Journal)	Present the new line of ReSound Nexia hearing aids and their technological features	Auracast is highlighted as a connectivity feature built into ReSound Nexia, enabling audio transmission in public environments and supporting more immersive and socially inclusive listening experiences
Humes et al.^(18)^	USA	Technical recommendation article – consensus report (scientific journal)	Propose a model for hearing well-being throughout adulthood	Auracast is mentioned as an emerging assistive technology that can contribute to short-term auditory rehabilitation, being listed among technologies that can be used together with magnetic induction systems, FM systems and closed captions
Kaufmann et al.^(2)^	USA	Conference paper (ArXiv preprint)	Investigate barriers to mass adoption of assistive listening technologies and propose viable technical solutions	Auracast is highlighted as a technology with the potential for wide adoption, offering high-fidelity stereo sound, with preserved environmental auditory perception (transparency mode) and compatibility with personal devices
Millett^(5)^	Canada	Review article (scientific journal)	Explore how auditory and visual access technologies can support children with hearing loss at home, school and in the community	Auracast is mentioned as a promising Bluetooth LE Audio innovation, with the potential to expand accessibility in public environments through direct connectivity and without additional devices
Puseman^(19)^	USA	Thesis	Determine the optimal separation distance between simultaneously transmitting Auracast devices, based on transmission failures and signal strength	Two to three devices must be separated by up to 13.7 meters, and four to six by up to 9.1 meters, to keep the signal above −62 dBm* and avoid transmission failures, optimizing reliability in environments with multiple simultaneous transmissions
Sterkens and Whyman^(20)^	Canada	Technical opinion article (scientific journal)	Reflect on the current assistive technology and the future of Auracast adoption compared to telecoil listening systems	Auracast is recognized as an emerging technology for assistive audio transmission, but its widespread implementation still depends on international standards, overcoming latency delays, simplifying access via intermediary devices and greater compatibility with hearing aids
Sterkens^(21)^	USA	Technical document (requirements guide)	Detail the technical, practical and regulatory requirements for Auracast to be viable as an assistive listening system	Widespread adoption depends on international standardization, compatibility with hearing instruments, technical integration with existing systems, and overcoming usability barriers
Sterkens^(22)^	USA	Technical document (technical guide)	Present practical guidelines for consumers when choosing assistive listening systems in public environments	Technology still under development for use as an assistive listening system, with limitations such as the need for a smartphone, dependence on applications and the absence of an IEC standard until 2027
Stucki and Zahnd^(23)^	Switzerland	Thesis	Develop a test infrastructure for LE Audio and Auracast-enabled devices, extending the Sonova Holding AG test environment	Creation of a functional test environment for Auracast transmitters, validating signal reception and processing
Sygrove^(24)^	USA	Technology article (online dissemination)	Introduce Auracast as a new Bluetooth connectivity technology for hearing aids	Emerging technology that allows hearing devices to receive audio transmissions directly from a variety of sources, providing a personalized and immersive listening experience

* dBm is a unit that expresses signal power in decibels in relation to 1 milliwatt

Most publications portray Auracast as a technological innovation with high potential impact on the hearing experience of people with hearing loss. Analyses highlight advantages such as high sound quality, low latency, compatibility with multiple devices, simultaneous transmission to different receivers, and integration into public spaces. However, the documents also emphasize important challenges, such as the need for international standardization, adequate infrastructure, usability barriers, and ensuring security and interoperability.

The records included address the applicability of Auracast in various contexts. In educational settings, for example, Millett^(5)^ highlights the potential of the technology to support students from early childhood education to higher education in situations involving noise, reverberation, and distance. In the field of culture and entertainment, Bailey^(11)^ describes the pioneering implementation of Auracast at the Sydney Opera House, demonstrating its value in live performances. Furthermore, technical documents, such as Sterkens^(22)^, explore the coexistence of Auracast with systems like telecoil and FM, promoting a more inclusive and accessible ecosystem.

Regarding the implementation forecast (Table 2), it is observed that most publications do not present defined deadlines. However, Sterkens^(21,22)^ and Sterkens & Whyman^(20)^ show that large-scale adoption will depend on the publication of the IEC 60118-17 standard, scheduled for December 2027. Puseman^(19)^ estimates that Auracast will be widely present in public spaces by 2030. Sources such as GN Hearing^(17)^ and CNET^(10)^ indicate that the technology is already being incorporated into hearing aids and Android smartphones, although full use still depends on compatibility between devices. Further information on the characteristics of the publications included in the review are shown in Table 1.

**Table 2:** Auracast implementation forecast

**Authors**	**Implementation forecast**	**Notes**
Bailey^(11)^	Start of adoption in 2025	Pioneering installation at the Sydney Opera House; demonstrates viability but is still an isolated case
Bruce et al.^(12)^	Not specified	It points out technical limitations that are still open (latency, volume calibration), with no estimated deadline for full adoption
Al-Heeti^(10)^	In progress since 2024	Auracast is available on Android 15; practical implementation depends on compatibility with hearing aids
Ceccato et al.^(13)^	Not specified	It mentions Auracast potential for public environments; it does not mention an implementation schedule
Edwards et al.^(14)^	Not specified	Focus on computer connectivity; no forecast for adoption as a large-scale assistive listening system
Fabry^(15)^	Not specified	Points out Auracast revolutionary potential; no set deadlines for widespread adoption
Gasteiger et al.^(16)^	Not specified	BACON is a proposed authentication solution for BISes; still in the experimental phase and with no timeline for commercial adoption
GN Hearing^(17)^	In progress since 2024	Auracast already integrated into the ReSound Nexia line; adoption still limited to compatible devices
Humes et al.^(18)^	Not specified	Auracast is listed among emerging technologies, but without temporal projections of practical use as an assistive listening system
Kaufmann et al.^(2)^	Not specified	Emerging technology, but still with critical technical issues such as automatic connectivity and acoustic delay
Millett^(5)^	Not specified	Points to Auracast as promising but does not detail the adoption timeline
Puseman^(19)^	By 2030	Auracast is expected to be widely available in public environments such as airports, churches, cinemas and classrooms by the end of the decade
Sterkens and Whyman^(20)^	After 2027	No permanent public installations with Auracast registered by 2025; wide use will depend on the supported standard and infrastructure
Sterkens^(21)^	After 2027	The IEC 60118-17 standard will only be published in December 2027; full adoption depends on this standardization
Sterkens^(22)^	After 2027	The IEC 60118-17 standard will only be published in December 2027; full adoption depends on this standardization
Stucki and Zahnd^(23)^	Not specified	Although support is still limited, some brands have already adopted Auracast and progressive expansion is expected in the near future
Sygrove^(24)^	Not specified	Presents Auracast as an emerging technology; does not mention implementation forecast

The combined analysis of the sources included show that, although Auracast is already being tested and adopted in some specific contexts, its full and effective adoption as an assistive listening system is still underway, depending on technical regulations, infrastructure development, and acceptance by the market and end users.

## DISCUSSION

This scoping review identified an emerging and diverse body of publications that point to the transformative potential of Auracast as an assistive technology. The publications reviewed highlight its ability to promote a more accessible, personalized, and immersive hearing experience, especially in public settings such as schools, theaters, museums, conference centers, and transportation systems^( 5,11,13)^.

Although practical initiatives already exist, such as the pioneering implementation at the Sydney Opera House^(11)^, the full adoption of Auracast still depends on structural and regulatory factors. Among the main challenges are the need for both the public environment and the user to have devices compatible with the technology - which requires financial investments in modern transmitters and DEAs^(17,21)^. Furthermore, the lack of a definitive international standardization (such as the IEC 60118-17 standard, expected only in December 2027) limits the full integration of the technology^(20,22)^. Despite this, pilot experiences such as the deployment of the Auri™ system at Bristol Temple Meads station in the United Kingdom confirm the interest in evaluating the scalability of Auracast in public transport environments, with a view to future expansion throughout the British rail network^(25)^.

Energy efficiency and sound quality are also frequently highlighted points. According to technical interviews^(24)^, Auracast stands out for consuming less energy and offering high-fidelity, low-latency stereo audio - essential characteristics for end-user comfort and acceptance.

Concerns about the security of data transmission have led to the proposal of solutions such as the BACON protocol, aimed at authenticating devices on Auracast networks to mitigate vulnerabilities^(16)^. Although still experimental, this proposal reinforces the need to ensure the integrity and reliability of connections in public environments.

In the educational context, studies such as those by Millett^(5)^ and Humes et al.^(18)^ highlight the potential of Auracast to expand the auditory accessibility of students from early childhood education to higher education. However, its effective implementation requires educators and caregivers to master the technology's configurations, and institutions to have compatible infrastructure. It should also be noted that, to date, Auracast-enabled hearing aids are not available in pediatric lines, limiting their application in younger populations. Despite these challenges, recent uses at events such as HLAA 2024, where conference rooms were equipped with Auracast transmitters to accommodate a wide variety of users with different hearing aids, indicate progress in the technical feasibility and visibility of the technology in learning environments^(26)^.

In general, publications are still recent and heterogeneous in their approach, but they converge in pointing out that Auracast represents a significant advance in the field of hearing accessibility. Its consolidation as an assistive listening system will depend on public policies, industry actions, and greater dissemination of technical knowledge among users and hearing health professionals.

### Limitations

During the course of this review, a significant scarcity of consolidated scientific publications directly addressing the application of Auracast in clinical, educational, or community settings was observed. Many of the available sources still focus on preliminary technical aspects or originate from conferences, manufacturers, and outreach content. This limitation hindered the construction of a more robust analysis based on clinical evidence.

### Recommendations

Further studies are recommended to explore the application of Auracast in educational and healthcare settings, its interactions with other assistive technologies (such as digital remote microphone systems and telephone coils), and its effectiveness across different population profiles. Based on the results of these initial investigations, it will be possible to more thoroughly assess the conditions necessary for the future large-scale adoption of the technology - considering technical, financial, and social aspects, especially in contexts with limited infrastructure.

## CONCLUSION

This scoping review made it possible to identify and systematize existing publications that address Auracast technology in its interface with hearing aids, highlighting its potential to promote hearing accessibility in public environments. The sources analyzed reinforce that Auracast represents an emerging technological solution among assistive technologies, bringing together features such as efficient connectivity, low energy consumption, high-quality audio transmission, and compatibility with multiple devices - aspects that position it as a relevant solution for the future of hearing accessibility.

The adoption of this technology in spaces such as schools, museums, transportation stations, and cultural centers can significantly expand access to communication in public environments for people with hearing loss. However, large-scale implementation still depends on regulatory factors, infrastructure investments, and the renewal of compatible hearing aids - currently unavailable in pediatric lines, for example. Furthermore, a large portion of the reviewed publications are technical, descriptive, or opinion-based, predominantly institutional documents, guides, and conference presentations. There is a significant gap in clinical or experimental studies with strong scientific evidence evaluating the effectiveness of Auracast in improving hearing, reducing auditory effort, or increasing adherence to the use of hearing aids in real-world contexts.

In addition to the recent introduction of the technology, the fact that technical standards are still under development - with publication expected only in 2027 - limits the maturity of the field and highlights its emerging nature. This scenario points to the urgency of applied, controlled, and long-term studies that rigorously evaluate the benefits and challenges of adopting Auracast as an assistive listening system.

Assistive technologies remain essential to ensuring autonomy, equity, and inclusion for people with hearing loss, and the development of solutions like Auracast represents a significant step forward in this direction, even though it is still undergoing scientific validation and practical consolidation.
