# Supplementary material for: Auracast and hearing devices: a scoping review on connectivity and accessibility for individuals with hearing loss in public environments
Source: Codas. 2026 Jul 6;38(4):e20250241. doi: 10.1590/2317-1782/e20250241en (PMC13343787; doi:10.1590/2317-1782/e20250241en)
Supplement: Translation

# Translation: Auracast e dispositivos auditivos: uma revisão de escopo sobre conectividade e acessibilidade para indivíduos com perda auditiva em ambientes públicos

**DOI:** 10.1590/2317-1782/e20250241pt

**Authors:** Lucas Marini Gonçalves, Anderson Vinícius de Moraes Ortega, Thais Corina Said de Angelo, Adriane Lima Mortari Moret, Natália Barreto Frederigue Lopes, Regina Tangerino de Souza Jacob

**Affiliations:** 1 Departamento de Fonoaudiologia, Faculdade de Odontologia de Bauru – FOB, Universidade de São Paulo – USP - Bauru (SP), Brasil.

**Keywords:** Tecnologia Assistiva, Aparelhos Auditivos, Acessibilidade para Pessoas com Deficiência, Educação Inclusiva, Comunicação Bluetooth

## Abstract

**Objetivo:**

Mapear e sintetizar as evidências disponíveis sobre o uso da tecnologia Auracast em dispositivos eletrônicos de amplificação sonora (DEAS), no contexto da acessibilidade auditiva em ambientes públicos.

**Estratégia de pesquisa:**

A revisão seguiu as diretrizes do Joanna Briggs Institute (JBI) e do PRISMA-ScR. Foi utilizada a estrutura PCC (População: indivíduos com perda auditiva usuários de DEAS; Conceito: tecnologia Auracast; Contexto: ambientes públicos). A busca foi realizada em abril de 2025 e abrangeu o período de junho de 2022 a abril de 2025, utilizando o termo “Auracast” como palavra-chave. As fontes de informação incluíram bases de dados científicas (PubMed, Scopus, IEEE Xplore, Dimensions Analytics) e fontes de literatura cinzenta (Google Acadêmico, sites de fabricantes de DEAS, associações profissionais e repositórios institucionais, como o OSF e o OpenGrey).

**Critérios de seleção:**

A seleção e extração dos dados foram realizadas por dois revisores independentes e cegados, com resolução de conflitos por consenso.

**Análise dos dados:**

Foram identificados 348 registros. Após a remoção de duplicatas e a aplicação dos critérios de elegibilidade, 17 publicações foram incluídas na síntese final.

**Resultados:**

As evidências disponíveis apontam para o potencial do Auracast em promover maior acessibilidade e autonomia em espaços coletivos, especialmente quando integrado a DEAS. Entretanto, as publicações encontradas são predominantemente técnicas ou descritivas, com escassez de avaliações clínicas robustas.

**Conclusão:**

O Auracast representa uma inovação emergente no campo da acessibilidade auditiva, mas ainda são necessários estudos empíricos que avaliem sua eficácia em ambientes educacionais, de saúde e de transporte. A adoção em larga escala dependerá de fatores técnicos, financeiros e sociais, especialmente em contextos com infraestrutura limitada.

## INTRODUÇÃO

Desde sua introdução nos dispositivos eletrônicos de amplificação sonora (DEAS), doravante denominados aparelhos auditivos, em 2005, a tecnologia Bluetooth tem desempenhado um papel fundamental na conectividade de indivíduos com perda auditiva, permitindo a transmissão direta de áudio para dispositivos como celulares, tablets e computadores^(1)^. Recentemente, uma inovação nesse campo emergiu: o Bluetooth Low Energy (LE) Audio com Auracast, um avanço projetado para integrar tecnologias de áudio sem fio e aprimorar a acessibilidade auditiva. Semelhante ao funcionamento das redes Wi-Fi, essa tecnologia permite que usuários com dispositivos compatíveis se conectem a sistemas de transmissão de áudio em ambientes equipados com Auracast^(2)^.

A tecnologia Auracast representa um avanço significativo na conectividade auditiva em espaços públicos. Equipamentos compatíveis, como TVs, celulares, notebooks e microfones, podem transmitir áudio diretamente para dispositivos receptores, incluindo aparelhos de amplificação sonora individual, fones de ouvido e smartwatches^(3)^. Esse recurso é particularmente relevante para indivíduos com perda auditiva, pois possibilita uma experiência sonora mais clara e inteligível, reduzindo o esforço auditivo e a fadiga associada à comunicação em ambientes ruidosos ou reverberantes. Além disso, a tecnologia viabiliza a transmissão de áudio em grande escala, permitindo sua aplicação em salas de aula, centros de conferências, estádios, igrejas e demais locais que demandam acessibilidade auditiva ampliada^(4)^.

O desenvolvimento de tecnologias assistivas tem sido essencial para promover a inclusão e melhorar a comunicação de pessoas com deficiência auditiva. A rede Auracast, ao oferecer transmissão sem fio de áudio sem restrições quanto ao número de dispositivos conectados, apresenta uma alternativa tecnológica relevante para a democratização do acesso à informação sonora. Sua implementação pode transformar a acessibilidade auditiva em espaços públicos e privados, representando um avanço substancial na conectividade sem fio para esse público^(5)^.

Diante desse cenário, este estudo tem como objetivo mapear e sintetizar as evidências científicas disponíveis sobre a tecnologia Auracast, baseada em Bluetooth Low Energy, aplicada aos DEAS, com foco em sua contribuição para a acessibilidade auditiva em ambientes públicos. Considerando o recente lançamento e a rápida disseminação do Auracast, a hipótese é de que já existam registros técnicos e científicos que demonstrem seu potencial de aplicação nos aparelhos auditivos para a promoção da acessibilidade auditiva em ambientes públicos, ainda que os estudos clínicos sejam escassos.

Com essa abordagem, espera-se contribuir significativamente para a evolução da conectividade sem fio em dispositivos auditivos, fornecendo suporte teórico e prático à academia, aos profissionais da saúde auditiva e aos formuladores de políticas públicas^(6)^.

## MÉTODO

Esta Revisão de Escopo foi desenvolvida com base na metodologia do *Joanna Briggs Institute (JBI) Reviewers’ Manual* e segue as diretrizes *PRISMA-ScR* (*Preferred Reporting Items for Systematic Reviews and Meta-Analyses Extension for Scoping Reviews*), garantindo transparência e reprodutibilidade^(7,8)^. O protocolo de pesquisa foi submetido para avaliação e registro na plataforma *Open Science Framework (OSF)* ^(9)^.

### Critérios de elegibilidade

Os critérios de elegibilidade foram definidos com base no modelo PCC (População, Conceito e Contexto), sendo: População — indivíduos com perda auditiva que utilizam DEAS; Conceito — tecnologia Auracast aplicada aos dispositivos auditivos; Contexto — acessibilidade auditiva em ambientes públicos.

### Critérios de inclusão e exclusão

Os critérios de inclusão foram definidos com base no modelo PCC, considerando publicações que abordassem indivíduos com perda auditiva que utilizassem DEAS, que tratassem explicitamente da tecnologia Auracast em contextos de acessibilidade auditiva em ambientes públicos, como escolas, centros culturais, igrejas, estações de transporte e outros espaços coletivos. Foram incluídos documentos publicados entre junho de 2022 (data de lançamento oficial do Auracast) e abril de 2025, disponíveis em texto completo, de acesso gratuito e redigidos em português, inglês ou espanhol. A revisão contemplou diferentes tipos de publicações, como artigos científicos, dissertações, teses, relatórios técnicos, publicações institucionais e anais de eventos acadêmicos.

Foram excluídas publicações que não mencionavam o termo “Auracast” ou o abordavam de forma superficial, sem relação direta com a aplicação da tecnologia em aparelhos auditivos. Também foram excluídos documentos que focassem exclusivamente em outras tecnologias assistivas baseadas em Bluetooth, sem referência ao Auracast, publicações duplicadas, trabalhos indisponíveis na íntegra ou redigidos em idiomas não contemplados.

### Fontes de pesquisa e estratégia de busca

Para a identificação de fontes relevantes, utilizou-se exclusivamente o termo “Auracast” como palavra-chave em todas as fontes de informação. Embora não tenham sido empregados descritores controlados (como MeSH ou DeCS), nem termos livres associados ao conceito mais amplo de tecnologias assistivas, essa escolha se justifica pelo foco específico e delimitado da revisão na tecnologia Auracast, cujo nome é distintivo e recente, com ocorrência bem definida nas publicações.

As buscas foram realizadas nas seguintes bases: PubMed, BVS, SciELO, IEEE Xplore, ACM Digital Library, Cochrane Library e Scopus. Como o número de resultados nessas bases foi limitado, a busca foi ampliada para a plataforma Dimensions Analytics, que agrega diversas fontes de produção científica, técnica e institucional, e para o Google Acadêmico, considerado uma fonte de informação ampla, embora não classificado formalmente como base de dados.

Complementarmente, realizou-se uma busca manual direcionada à localização de literatura branca e cinzenta. Foram consultados repositórios institucionais (como OSF e OpenGrey), sites de fabricantes de DEAS (como GN Hearing e Sonova), e de associações setoriais relevantes (como o Bluetooth Special Interest Group – SIG e a Hearing Loss Association of America). Essa busca foi realizada por meio de navegação estruturada e do uso de expressões específicas nos mecanismos internos de busca ou em buscadores gerais.

A base Web of Science não foi incluída na estratégia de busca, uma vez que o delineamento metodológico priorizou bases com maior aderência simultânea às áreas de saúde, engenharia e tecnologia aplicada, como Scopus, IEEE Xplore, ACM Digital Library e Dimensions Analytics. Essas plataformas apresentam ampla cobertura multidisciplinar e forte indexação de publicações técnicas e de inovação tecnológica, consideradas particularmente relevantes para a temática do Auracast. Dessa forma, a estratégia adotada buscou maximizar a sensibilidade para identificar registros técnicos, científicos e institucionais relacionados à tecnologia, mantendo coerência com o caráter emergente e interdisciplinar do objeto investigado.

Foram considerados elegíveis documentos técnicos, guias, relatórios, comunicados institucionais, artigos científicos, dissertações, teses e anais de eventos. Dois revisores independentes realizaram a triagem dos materiais com base nos critérios estabelecidos, priorizando publicações que abordassem a aplicação do Auracast em aparelhos auditivos, com foco na acessibilidade auditiva.

Essa abordagem buscou contornar a escassez de evidências revisadas por pares, esperada diante da recente introdução oficial do Auracast pelo Bluetooth SIG em junho de 2022. Assim, a incorporação de literatura técnica e institucional foi essencial para garantir a abrangência e atualidade da revisão.

### Extração e armazenamento dos dados

Não foi utilizado um gerenciador de referências bibliográficas automatizado neste estudo. A organização e o controle das fontes foram realizados manualmente por meio de planilhas eletrônicas, que permitiram a triagem, a categorização e a exclusão de duplicatas de forma sistemática. A eliminação de referências duplicadas foi realizada por meio da comparação direta de títulos, autores, anos e links de acesso, com revisão cruzada entre os revisores. Embora ferramentas como Zotero ou Mendeley possam facilitar esse processo, optou-se por um controle direto e personalizado devido ao volume de registros manejável e à especificidade do termo “Auracast”, o que permitiu uma gestão precisa das publicações, sem prejuízo da transparência e da reprodutibilidade da revisão.

O processo de triagem foi conduzido em três fases: leitura de títulos, resumos e leitura completa dos textos selecionados.

Todas as etapas do processo de seleção e extração foram conduzidas de forma independente por dois revisores. Para garantir o cegamento, os registros foram organizados em planilhas sem identificação de autores, instituições ou periódicos. O acesso aos textos completos foi estruturado por código, e as planilhas de triagem foram compartilhadas separadamente com cada revisor. O cegamento foi mantido também na etapa de extração dos dados, que seguiu um protocolo pré-definido com campos padronizados.

Em caso de discordância entre os revisores — tanto nas fases de triagem (fase 1: leitura de títulos e resumos; fase 2: leitura completa) quanto na etapa de extração —, os conflitos foram discutidos em reunião conjunta. Quando não havia consenso, a decisão foi submetida a um terceiro revisor com experiência na temática, que avaliou os critérios de inclusão à luz do protocolo previamente definido.

Os dados extraídos foram analisados por dois autores, sendo categorizados em: Autores (ano), País, Classificação da Publicação e Observações Relacionadas ao Auracast.

### Análise e apresentação dos resultados

Os dados foram analisados de forma descritiva e foram apresentados por meio de figuras e tabelas, conforme recomendado por PRISMA-ScR^(8)^.

## RESULTADOS

### Revisão e seleção dos resultados

A busca inicial identificou um total de 348 registros, distribuídos nas seguintes fontes: Google Acadêmico (296), Dimensions Analytics (44), IEEE Xplore (2), PubMed (2), Scopus (4), SciELO (0), BVS (0), ACM Digital Library (0), Cochrane Library (0). A base Dimensions foi utilizada como alternativa para ampliar a cobertura da literatura técnica e científica relevante sobre o Auracast, dado o número reduzido de resultados em bases de dados convencionais. O Google Acadêmico foi incluído como fonte de informação para abranger materiais não indexados em bases tradicionais, conforme recomendado para revisões que envolvem literatura cinzenta.

Após a remoção manual de sete registros duplicados, 341 publicações seguiram para a etapa de triagem. Nesta fase, os títulos e resumos foram avaliados com base nos critérios PCC: a exclusão de 322 registros ocorreu por não abordarem a tecnologia Auracast no contexto de acessibilidade auditiva, por tratar-se de tecnologias distintas ou por se referirem a áreas técnicas não relacionadas à população-alvo (indivíduos com perda auditiva usuários de DEAS) ou ao contexto de ambientes públicos.

Das 19 publicações selecionadas para leitura na íntegra, duas foram excluídas por indisponibilidade de acesso gratuito ao texto completo. Embora tenha sido realizada uma tentativa de contato com os autores, não houve retorno no prazo definido para a extração. Dessa forma, 17 documentos atenderam a todos os critérios de elegibilidade e foram incluídos na síntese final. O fluxograma completo do processo de seleção das publicações pode ser consultado na Figura 1, conforme as diretrizes do modelo PRISMA-ScR.

**Figura 1:** Fluxo do processo de seleção das publicações incluídas na revisão, conforme as etapas de identificação, triagem, elegibilidade e inclusão, com base no modelo PRISMA-ScR

### Características do estudo

Os 17 estudos selecionados para esta revisão foram divulgados entre 2023 e 2025, em formatos diversos — como artigos científicos, teses, documentos técnicos, comunicados de fabricantes e conteúdos de divulgação online — e publicados majoritariamente em inglês, com uma exceção em francês (também traduzida para o espanhol) (Tabela 1). A diversidade de fontes evidencia o interesse crescente por tecnologias emergentes aplicadas à acessibilidade auditiva.

**Tabela 1:** Síntese dos resultados

**Autores**	**País**	**Classificação da Publicação**	**Objetivo do Estudo**	**Observações Relacionadas ao Auracast**
Al-Heeti^(10)^	EUA	Artigo de tecnologia (divulgação online)	Anunciar a integração do suporte ao Auracast no Android, permitindo que dispositivos eletrônicos de amplificação sonora se conectem diretamente a transmissões de áudio	Auracast é adicionado ao Android, permitindo que dispositivos eletrônicos de amplificação sonora se conectem diretamente a transmissões de áudio, como anúncios públicos, melhorando significativamente a acessibilidade auditiva para usuários com deficiência auditiva
Bailey^(11)^	Austrália	Artigo de tecnologia (divulgação online)	Relatar a implementação do Auracast no Sydney Opera House e discutir suas vantagens em relação aos sistemas de escuta assistiva tradicionais	Auracast é implantado de forma pioneira no Sydney Opera House, demonstrando sua instalação rápida e acessível, proporcionando áudio de alta qualidade sem interferências, superando limitações dos sistemas de escuta assistiva baseados em indução magnética
Bruce et al.^(12)^	Canadá/ Índia	Artigo em conferência (ICASSP)	Analisar o desenvolvimento do padrão Bluetooth LE Audio com Auracast e discutir oportunidades e desafios técnicos para sua implementação como sistema de escuta assistiva	Auracast é apresentado como uma evolução tecnológica com potencial para substituir sistemas tradicionais de escuta assistiva, ao permitir transmissão de áudio estéreo com baixa latência e conexão universal a dispositivos eletrônicos de amplificação sonora e fones de ouvido, além de suportar múltiplos fluxos simultâneos — como diferentes idiomas, mixagens sonoras ou fontes específicas em ambientes públicos; no entanto, sua adoção ainda exige soluções para calibração de volume e compensação de atraso acústico
Ceccato et al.^(13)^	França	Artigo de revisão (periódico científico)	Descrever os fundamentos técnicos e clínicos da adaptação de dispositivos eletrônicos auditivos convencionais, incluindo estratégias de reabilitação auditiva	Auracast é apresentado como recurso tecnológico promissor, permitindo a transmissão simultânea de áudio para múltiplos dispositivos auditivos compatíveis, com potencial para ampliar a acessibilidade em ambientes públicos
Edwards et al. ^(14)^	EUA	Artigo em conferência (EPIC Proceedings)	Investigar as experiências de usuários de aparelhos de amplificação sonora individual ao conectar-se a PCs e o impacto nas decisões de design acessível	Auracast é citado como funcionalidade do Bluetooth LE Audio com potencial para superar barreiras técnicas de conectividade, permitindo pareamento simultâneo e eficiente entre dispositivos eletrônicos de amplificação sonora e PCs
Fabry ^(15)^	EUA	Artigo de divulgação profissional (ASHA Leader)	Explorar as inovações tecnológicas em dispositivos eletrônicos de amplificação sonora, com foco em conectividade e transmissão de áudio	Auracast é destacado como uma tecnologia emergente que, ao permitir transmissões de áudio para múltiplos dispositivos simultaneamente, promete revolucionar a experiência auditiva em ambientes públicos e privados, embora sua implementação ainda dependa de avanços em infraestrutura e compatibilidade de dispositivos
Gasteiger et al. ^(16)^	Áustria / Itália	Artigo em conferência (MILCOM)	Propor e validar um mecanismo de autenticação (BACON) para transmissões Auracast em ambientes públicos vulneráveis a ataques	Auracast é descrito com potencial para aplicações públicas em larga escala, mas sua adoção segura ainda requer soluções de autenticação como o BACON para mitigar vulnerabilidades e garantir integridade nas transmissões
GN Hearing ^(17)^	Dinamarca / EUA	Nota editorial em revista profissional (Manufacturers News – The Hearing Journal)	Apresentar a nova linha de dispositivos eletrônicos de amplificação sonora ReSound Nexia e suas funcionalidades tecnológicas	Auracast é destacado como recurso de conectividade integrado ao ReSound Nexia, permitindo transmissão de áudio em ambientes públicos e oferecendo suporte a experiências auditivas mais imersivas e socialmente inclusivas
Humes et al. ^(18)^	EUA	Artigo técnico de recomendação - relatório de consenso (periódico científico)	Propor um modelo para bem-estar auditivo ao longo da vida adulta	Auracast é citado como uma tecnologia assistiva emergente que pode contribuir para a reabilitação auditiva de curto prazo, listando-o como uma das tecnologias assistivas que podem ser utilizadas junto com sistemas de indução por aro magnético, sistemas FM e legendagem
Kaufmann et al. ^(2)^	EUA	Artigo de conferência (pré-print ArXiv)	Investigar as barreiras à adoção em massa de tecnologias assistivas de escuta e propor soluções técnicas viáveis.	Auracast é destacado como uma tecnologia com potencial para ampla adoção, oferecendo som estéreo de alta fidelidade, com percepção auditiva ambiental preservada (modo de transparência) e compatibilidade com dispositivos pessoais
Millett ^(5)^	Canadá	Artigo de revisão (periódico científico)	Explorar como tecnologias de acesso auditivo e visual podem apoiar crianças com perda auditiva em casa, na escola e na comunidade	Auracast é citado como inovação promissora do Bluetooth LE Audio, com potencial para ampliar a acessibilidade em espaços públicos por meio de conectividade direta e sem dispositivos adicionais
Puseman ^(19)^	EUA	Tese	Determinar a distância ideal de separação entre dispositivos Auracast transmitindo simultaneamente, com base em falhas de transmissão e intensidade de sinal	A publicação indica que dois a três dispositivos devem ser separados por até 13,7 metros, e quatro a seis por até 9,1 metros, para manter o sinal acima de -62 dBm^*^ e evitar falhas de transmissão, otimizando a confiabilidade em ambientes com múltiplas transmissões simultâneas
Sterkens e Whyman^(20)^	Canadá	Artigo técnico de opinião (periódico científico)	Refletir sobre o estado atual da tecnologia assistiva e o futuro da adoção do Auracast em comparação aos sistemas de escuta com telecoil	Auracast é reconhecido como tecnologia emergente para transmissão de áudio assistivo, mas sua implementação ampla ainda depende de padronizações internacionais, superação de atrasos de latência, simplificação do acesso via dispositivos intermediários e maior compatibilidade com aparelhos de amplificação sonora individual
Sterkens^(21)^	EUA	Documento técnico (guia de requisitos)	Detalhar os requisitos técnicos, práticos e regulatórios para que o Auracast seja viável como sistema de escuta assistiva	Auracast é descrito como uma tecnologia emergente para escuta assistiva em ambientes públicos, mas sua adoção ampla depende de padronização internacional, compatibilidade com instrumentos auditivos, integração técnica com sistemas existentes e superação de barreiras de usabilidade, como necessidade de dispositivos intermediários
Sterkens^(22)^	EUA	Documento técnico (guia técnico)	Apresentar diretrizes práticas para consumidores na escolha de sistemas de escuta assistiva em ambientes públicos	Auracast é classificado como uma tecnologia ainda em desenvolvimento para uso como sistema de escuta assistiva, destacando limitações como necessidade de smartphone, dependência de apps, e ausência de padrão IEC até 2027
Stucki e Zahnd ^(23)^	Suíça	Tese	Desenvolver uma infraestrutura de testes para dispositivos com LE Audio e Auracast, estendendo o ambiente de testes da Sonova Holding AG	Descreve a criação de um ambiente de testes funcional para transmissores Auracast, validando a recepção e o processamento dos sinais; destaca-se como iniciativa inicial para integrar a tecnologia nos processos da indústria
Sygrove^(24)^	EUA	Artigo de tecnologia (divulgação online)	Apresentar o Auracast como uma nova tecnologia de conectividade Bluetooth para dispositivos eletrônicos de amplificação sonora	Auracast é apresentado como uma tecnologia emergente de conectividade Bluetooth que permite que dispositivos eletrônicos de amplificação sonora recebam transmissões de áudio de diversas fontes, como TVs e sistemas de anúncio público, diretamente em seus dispositivos, proporcionando uma experiência auditiva personalizada e imersiva

* dBm é uma unidade que expressa a potência do sinal em decibéis em relação a 1 miliwatt

A maioria das publicações destaca o Auracast como uma inovação tecnológica com alto potencial de impacto na experiência auditiva de pessoas com perda auditiva. As análises apontam vantagens como alta qualidade sonora, baixa latência, compatibilidade com múltiplos dispositivos, transmissão simultânea para diferentes receptores e integração em espaços públicos. No entanto, os documentos também ressaltam desafios importantes, como a necessidade de padronização internacional, de infraestrutura adequada, de barreiras de usabilidade e de garantias de segurança e interoperabilidade.

Os registros incluídos abordam a aplicabilidade do Auracast em diversos contextos. Em ambientes educacionais, por exemplo, Millett^(5)^ destaca o potencial da tecnologia para apoiar alunos da educação infantil ao ensino superior em situações de ruído, reverberação e distância. No campo da cultura e do entretenimento, Bailey^(11)^ descreve a implementação pioneira do Auracast no Sydney Opera House, demonstrando seu valor em performances ao vivo. Além disso, documentos técnicos, como o de Sterkens^(22)^, exploram a coexistência do Auracast com sistemas como telecoil e FM, promovendo um ecossistema mais inclusivo e acessível.

Em relação à previsão de implementação (Tabela 2), observa-se que a maioria das publicações não apresenta prazos definidos. Entretanto, Sterkens^(21,22)^ e Sterkens e Whyman^(20)^ apontam que a adoção em larga escala dependerá da publicação da norma IEC 60118-17, prevista para dezembro de 2027. Já Puseman^(19)^ estima que o Auracast esteja amplamente presente em espaços públicos até 2030. Por sua vez, fontes como GN Hearing^(17)^ e CNET^(10)^ indicam que a tecnologia já está sendo incorporada por dispositivos auditivos e por smartphones Android, embora o uso pleno ainda dependa da compatibilidade entre os equipamentos. Mais informações sobre as características das publicações incluídas na revisão podem ser encontradas na Tabela 1.

**Tabela 2:** Previsões temporais para implementação do Auracast

**Autores**	**Previsão de Implementação**	**Observações**
Bailey ^(11)^	Início da adoção em 2025	Instalação pioneira no Sydney Opera House; demonstra viabilidade, mas ainda é um caso isolado.
Bruce et al. ^(12)^	Não especificada	Aponta limitações técnicas ainda em aberto (latência, calibração de volume), sem prazo estimado para adoção plena.
Al-Heeti ^(10)^	Em andamento desde 2024	Auracast está disponível no Android 15; a implementação prática depende da compatibilidade com dispositivos de amplificação sonora.
Ceccato et al.^(13)^	Não especificada	Cita o potencial do Auracast para espaços públicos; não há menção a cronograma de implementação.
Edwards et al.^(14)^	Não especificada	Foca em conectividade com computadores; não apresenta previsão de adoção como ALS em larga escala.
Fabry^(15)^	Não especificada	Aponta potencial revolucionário do Auracast; não há prazos definidos para adoção ampla.
Gasteiger et al.^(16)^	Não especificada	BACON é uma proposta de solução de autenticação em BISes; ainda em fase experimental e sem cronograma de adoção comercial.
GN Hearing^(17)^	Em andamento desde 2024	Auracast já integrado à linha ReSound Nexia; adoção ainda limitada a dispositivos compatíveis.
Humes et al.^(18)^	Não especificada	Auracast está listado entre as tecnologias emergentes, mas sem projeções temporais de uso prático, como ALS.
Kaufmann et al.^(2)^	Não especificada	Tecnologia emergente, mas ainda com problemas técnicos críticos (como a conectividade automática e o atraso acústico).
Millett^(5)^	Não especificada	Aponta o Auracast como promissor, mas não detalha o cronograma de adoção.
Puseman^(19)^	Até 2030	Estima-se que o Auracast esteja amplamente disponível em locais públicos, como aeroportos, igrejas, cinemas e salas de aula, até o final da década.
Sterkens e Whyman^(20)^	Após 2027	Nenhuma instalação pública permanente com Auracast registrada até 2025; o uso amplo dependerá da norma e da infraestrutura compatíveis.
Sterkens^(21)^	Após 2027	A norma IEC 60118-17 será publicada apenas em dezembro de 2027; a adoção plena depende dessa padronização.
Sterkens^(22)^	Após 2027	A norma IEC 60118-17 será publicada apenas em dezembro de 2027; a adoção plena depende dessa padronização.
Stucki e Zahnd^(23)^	Não especificada	Apesar do suporte ainda ser limitado, algumas marcas já adotaram o Auracast e espera-se uma expansão progressiva no futuro próximo.
Sygrove^(24)^	Não especificada	Apresenta o Auracast como uma tecnologia emergente; não menciona estimativas temporais.

A análise combinada das fontes incluídas revela que, embora o Auracast já esteja sendo testado e adotado em alguns contextos específicos, sua adoção ampla e efetiva como sistema de escuta assistiva ainda está em construção, dependendo de regulamentações técnicas, da evolução da infraestrutura e da aceitação pelo mercado e pelos usuários finais.

## DISCUSSÃO

Esta revisão de escopo identificou um conjunto emergente e diversificado de publicações que apontam para o potencial transformador do Auracast como tecnologia assistiva. As publicações revisadas destacam sua capacidade de promover uma experiência auditiva mais acessível, personalizada e imersiva, especialmente em ambientes públicos como escolas, teatros, museus, centros de conferência e sistemas de transporte^(5,11,13)^.

Embora já existam iniciativas práticas, como a implementação pioneira no Sydney Opera House^(11)^, a adoção ampla do Auracast ainda depende de fatores estruturais e regulatórios. Entre os principais desafios estão a necessidade de que tanto o ambiente público quanto o usuário possuam dispositivos compatíveis com a tecnologia — o que exige investimentos financeiros em transmissores e DEAS modernos^(17,21)^. Além disso, a ausência de uma padronização internacional definitiva (como a norma IEC 60118-17, prevista apenas para dezembro de 2027) limita a integração plena da tecnologia^(20,22)^. Ainda assim, experiências-piloto como a implantação do sistema Auri™ na estação Bristol Temple Meads, no Reino Unido, demonstram o interesse em avaliar a escalabilidade do Auracast em ambientes de transporte público, com vistas à futura expansão em toda a rede ferroviária britânica^(25)^.

A eficiência energética e a qualidade sonora também são pontos frequentemente ressaltados. De acordo com entrevistas técnicas^(24)^, o Auracast se diferencia por consumir menos energia e oferecer áudio estéreo de alta fidelidade e baixa latência — características essenciais para o conforto e a aceitação do usuário final.

A preocupação com a segurança dos dados transmitidos levou à proposição de soluções como o protocolo BACON, voltado à autenticação de dispositivos em redes Auracast para mitigar vulnerabilidades^(16)^. Ainda que experimental, essa proposta reforça a necessidade de garantir a integridade e a confiabilidade das conexões em ambientes públicos.

No contexto educacional, estudos como os de Millett^(5)^ e Humes et al.^(18)^ destacam o potencial do Auracast para ampliar a acessibilidade auditiva de estudantes desde a educação infantil até o ensino superior. Contudo, sua implementação efetiva exige que educadores e responsáveis dominem as configurações da tecnologia, além de depender da existência de infraestrutura compatível nas instituições. Ressalta-se, ainda, que, até o momento, os DEAS com suporte ao Auracast não estão disponíveis nas linhas pediátricas, o que limita sua aplicação em populações mais jovens. Apesar desses desafios, demonstrações recentes em eventos como a HLAA 2024, onde salas de conferência foram equipadas com transmissores Auracast para atender uma grande variedade de usuários com diferentes DEAS, indicam um avanço na viabilidade técnica e na visibilidade da tecnologia em ambientes de aprendizagem^(26)^.

De modo geral, as publicações ainda são recentes e heterogêneas quanto à abordagem, mas convergem ao apontar que o Auracast representa um avanço relevante no campo da acessibilidade auditiva. Sua consolidação como sistema de escuta assistiva dependerá de políticas públicas, ações da indústria e maior disseminação de conhecimento técnico entre os usuários e profissionais da saúde auditiva.

### Limitações

Durante a condução desta revisão, observou-se uma escassez significativa de publicações científicas consolidadas que abordem diretamente a aplicação do Auracast em contextos clínicos, educacionais ou comunitários. Muitas das fontes disponíveis ainda se concentram em aspectos técnicos preliminares ou são oriundas de conferências, fabricantes e de conteúdos de divulgação. Essa limitação dificultou a construção de uma análise mais robusta e baseada em evidências clínicas.

### Recomendações

Recomenda-se o desenvolvimento de novos estudos que explorem a aplicação do Auracast em ambientes educacionais e de saúde, suas interações com outras tecnologias assistivas (como sistemas de microfone remoto digital e bobinas telefônicas) e sua eficácia em diferentes perfis populacionais. A partir dos resultados dessas investigações iniciais, será possível avaliar, com maior embasamento, as condições necessárias para a adoção futura, em larga escala, da tecnologia — considerando aspectos técnicos, financeiros e sociais, especialmente em contextos com infraestrutura limitada.

## CONCLUSÃO

Esta revisão de escopo possibilitou identificar e sistematizar as publicações existentes que abordam a tecnologia Auracast em sua interface com DEAS, destacando seu potencial para promover acessibilidade auditiva em ambientes públicos. As fontes analisadas reforçam que o Auracast representa uma solução tecnológica emergente entre as tecnologias assistivas, reunindo características como conectividade eficiente, baixo consumo de energia, transmissão de áudio de alta qualidade e compatibilidade com múltiplos dispositivos — aspectos que a posicionam como uma solução relevante para o futuro da acessibilidade auditiva.

A adoção dessa tecnologia em espaços como escolas, museus, estações de transporte e centros culturais pode ampliar significativamente o acesso de pessoas com perda auditiva à comunicação em ambientes coletivos. No entanto, a implementação em larga escala ainda depende de fatores regulatórios, de investimentos em infraestrutura e da renovação de dispositivos auditivos compatíveis — atualmente indisponíveis em linhas pediátricas, por exemplo. Além disso, grande parte das publicações revisadas possui caráter técnico, descritivo ou opinativo, com predominância de documentos institucionais, guias e apresentações em conferências. Observa-se uma lacuna importante de estudos clínicos ou experimentais com forte evidência científica que avaliem a eficácia do Auracast na melhoria da escuta, na redução do esforço auditivo ou na adesão ao uso de DEAS em contextos reais.

Além da recente introdução da tecnologia, o fato de as normativas técnicas ainda estarem em fase de desenvolvimento — com previsão de publicação apenas em 2027 — limita a maturidade do campo e evidencia seu caráter emergente. Esse cenário destaca a urgência de estudos aplicados, controlados e de longo prazo que avaliem, de forma rigorosa, os benefícios e os desafios da adoção do Auracast como sistema de escuta assistiva.

As tecnologias assistivas permanecem essenciais para garantir autonomia, equidade e inclusão para pessoas com perda auditiva, e o desenvolvimento de soluções como o Auracast representa um avanço significativo nesse caminho, ainda que em processo de validação científica e de consolidação prática.
